# Nutlet micromorphology and character evolution of *Lappula* species (Boraginaceae) and its systematic implications

**DOI:** 10.1186/s40529-021-00325-6

**Published:** 2021-10-24

**Authors:** Mostafa Ebadi, Sedigheh Nikzat

**Affiliations:** 1 Department of Biology, Faculty of Sciences, Azarbaijan Shahid Madani University, 53714-161 Tabriz, Iran; 2 Faculty of Life Sciences & Biotechnology, Shahid Beheshti University, Tehran, Iran

**Keywords:** Character evolution, *Lappula*, Micromorphology, Systematic

## Abstract

**Background:**

The macro/micro-morphology of nutlets in 11 species (and 22 accessions) of the Boraginaceae family was investigated using stereomicroscope and scanning electron microscopy to evaluate the taxonomic relevance of the traits. To evaluate the phylogenetic significance of the character evolution, phylogenetic analysis was carried out by comparing available DNA sequence data from GenBank with selected original nutlet data.

**Results:**

The Rochelieae nutlets' shape varied from ovoid (ovoid, ovoid-triangular, and ovoid-rectangular) to pyramid. Six major patterns were recognized based on the nutlet ultrastructure characters. Rocheliae is characterized by a transition from “without appendage” to “with tubercles and prickles” on the nutlet disk, and also via a shift from “lack of prickles” to “glossy prickles”.

**Conclusions:**

The results show that the nutlet ultrastructure pattern of Rochelieae is systematically informative at the genus level, but not at the species level. Findings demonstrated that glochid is not an ancestral trait but is a synapomorphy and the transition to this trait occurred in the genus *Lappula*. The close boundary of nutlet microstructures between *L. barbata* and *L. microcarpa* has been discussed.

## Background

Boraginaceae s.str. is a sub cosmopolitan family of flowering plants with nearly 90 genera and ca.1600 to 1700 species distributed globally. According to a molecular phylogeny study by Chacón et al. (2016), infrafamilial classification of Boraginaceae might fall within three Subfamilies (Echiochiloideae, Boraginoideae, Cynoglossoideae) and 10 tribes (Boragineae, Lithospermeae, Trichodesmeae, Lasiocaryeae, Asperugeae, Omphalodeae, Rochelieae, Craniospermeae, Myosotideae, Cynoglosseae). Tribe Rochelieae consists of approximately 207 species, and belongs to the subfamily Cynoglossoideae. According to Chacón et al. (2016), Rochelieae can be divided into two subtribes including Eritrichiinae and Heterocaryinae. The genera *Eritrichium*, *Hackelia*, *Lappula*, *Lepechiniella,* and *Rochelia,* belong to the Eritrichiinae and the genera *Heterocaryum*, *Suchtelenia*, and *Pseudoheterocaryum* to sub-tribe Heterocaryinae (Chacón et al. 2016; Saadati et al. 2017). Three genera of subtribe Eritrichiinae (*Eritrichium*, *Lepechiniella,* and *Lappula*) are recognized as non-monophyletic lineage. *Hackelia* and *Rochelia* are comprised of monophyletic clades (Khoshsokhan-Mozaffar et al. 2018). The recent molecular phylogeny of the Rochelieae tribe provided by Khoshsokhan-Mozaffar et al. (2018) has indicated a well-supported clade. *Hackelia* and *Rochelia* are monophyletic while *Lappula, Eritrichium*, and *Lepechiniella* are not. Therefore, *Lappula,* as currently circumscribed, is polyphyletic (Khoshsokhan-Mozaffar et al. 2018). The genus *Eritrichium* is the largest genus of the tribe, with 71 species. The genus *Lappula* contains about 70 species of annual, biennial perennial herbs distributed in Eurasia, Africa, North America, and Australia (Ovchinnikova 2005). Although *Lappula* has a cosmopolitan distribution, the center of diversity is in the Siberian and Irano-Turaniean provinces of the Holarctic kingdom (Ovchinnikova 2009). Initially, Lehmann (1818) circumscribed 15 species in *Echinospermum* Lehm. (Synonym of *Lappula*). *Echinospermum* was divided by de Candolle (1845) into three sections based on nutlet morphology as follow: *Lappula*, *Sclerocaryum*, and *Homalocaryum*. The taxonomical problems of *Lappula* were increased as the number of species in the genus began to expand. Consequently, the number of sections, subsections, and series seems to be widely varied upon what has been reported by different authors (Popov 1953; Riedl 1967). First off, Lehmann (1818) used nutlet characters in the systematics of *Lappula* and showed mericarp characters including mericarp shape and surface ornamentation to distinguish the species of this genus. Generally, regarding Boraginaceae, nutlet morphology provides valid systematic characters at various taxonomic levels, such as straight or incurved nutlet, a specialized form of emergence, the position of attachment scar, the distinctive form of prickles or glochids, and epidermal features of nutlets (Johnston 1937; Hilger 1985; Al-Shehbaz 1991; Riedl 1996; Långström and Chase 2002; Moon and Hong 2006; Selvi et al. 2006; Kahraman et al. 2011). In this study, we aimed to evaluate the nutlet morphological characters of several Iranian *Lappula* species and compare them to related genera in Rochelieae by scanning samples using electron microscopy. DNA sequence data and selected nutlet characters were examined to investigate the character evolution and phylogenetic relationships. The results will be discussed with a particular focus on Iranian species of *Lappula* and some related genera of the Rochelieae tribe.

## Methods

### Morphological study

The plants used in the present study were collected from their natural habitats in Iran and deposited in the Herbarium of Azarbaijan Shahid Madani University (ASMUH). Also, a small number of species were taken from herbarium specimens of FUMH (Ferdowsi University of Mashhad Herbarium). The list of voucher specimens and details of their locations were given in Table 1.

**Table 1:** List of sampled taxa, locality and their vouchers numbers

Species (Pop. Code)	Locality	Voucher No.
*Lappula barbata* (M.Bieb.) Gürke (m)	Tehran, Chalus road, Kooshk	ASMUH0020
*L. barbata* (c)	Mazandaran, Chalus, Delir vilage	ASMUH0021
*L. barbata* (ab)	Mazandaran, Noshahr, Kojur, Laregan	ASMUH0022
*L. barbata* (w)	Tehran, Tuchal	ASMUH0023
*L. ceratophora* (Popov) Popov	South Khorasan, south-west Sarayan	FUMH46077
*L. microcarpa* (Ledeb.) Gürke (b)	Mazandaran. Noor, Chamestan, Lavij	ASMUH0024
*L. microcarpa* (e)	Mazandaran, Neka, Hezarjerib	ASMUH0025
*L. microcarpa* (g)	Golestan, East of Golestan national park	ASMUH0026
*L. microcarpa* (i)	Mazandaran, Savadkooh, Veresk	ASMUH0027
*L. microcarpa* (j)	Mazandaran, Polor to Rine	ASMUH0028
*L. microcarpa* (p)	Mazandaran, Noshahr, Kojur,	ASMUH0029
*L. microcarpa* (z)	North Khorasan, Chamanbid	ASMUH0030
*L. microcarpa* (a)	Tehran, Lavasan, Glucan	ASMUH0031
*L. semiglabra* (Ledeb.) Gürke	Khorasan Razavi, North of Gonabad	FUMH17236
*L. sessiliflora* (Boiss.) Gürke	Khorasan Razavi, East of Kashmar	FUMH26636
*L. spinocarpus* (Forssk.) Asch. ex Kuntze	South Khorasan, Birjand, Shahzile	FUMH30399
*Pseudolappula sinaica* (A.DC.) Asch. & Schweinf	Tehran, Chalus road, Morod	ASMUH0032
*Asperugo procumbens* L	Mazandaran, Damavand, Sarbandan	ASMUH0034
*Heterocaryum rigidum* A. DC	Tehran, Jajroad	ASMUH0035
*Myosotis sylvatica* Ehrh	Mazandaran, Sari, Sangdeh forest	ASMUH0036
*Rochelia disperma* (L. f) C. Koch	Tehran, Lavasan	ASMUH0037

This study was carried out on nine species of tribe Rochelieae (covering four genera) and two species of tribes Asperugeae and Myosotideae as out-groups. Depending on the amount of material available, 10 nutlets of each taxon were investigated and scored for the standard descriptors in Table 2. The air dried nutlets (10 per each taxon and accession) were investigated for their shape, size, and other features using stereomicroscope (Dino-Lite) using the DinoCapture eye and DinoCapture 2.0 Software (Electronics Corporation).

**Table 2:** Micromorphological characters of the nutlet in the studied taxa

Taxon	Characters								
	SN	CL	BPD	LS	AND	NGRN	TD	SE	PS
*L.microcarpa*	0	0	1	1	2	1	2	0	2
*L. barbata*	0	1	0	1	2	2	2	0	2
*L. semiglabra*	0	0	1	1	2	1	0	0	2
*L.ceratophora*	4	0	0	1	1	0	0	1	0
*L. spinocarpos*	4	0	0	1	1	0	0	1	0
*L. sessiliflora*	3	2	2	1	2	1	3	0	1
*R. disperma*	0	0	0	1	2	0	1	0	1
*L. siniaca*	0	0	0	3	0	0	0	2	0
*H. rigidum*	2	0	0	2	0	1	0	2	2
*A. procumbens*	1	0	0	0	3	0	0	3	0
*M. sylvatica*	5	0	0	1	1	0	0	4	0

SN (shape of nutlet) including ovoid (0), semicircular (1), ovoid-rectangular (2), ovoid-triangular (3), pyramid (4), ellipse (5); CL (center line of nutlet) including absent (0), prominent with glochid (1), prominent with tubercles (2); BPD (base surface of prickles on desk) including lack of prickles (0), glabrous (1), verrucose (2); LS (Lamella status) including thread like (0), glossy (1), bumpy with verrucate (2), interrupt (3); AND (Appendage on nutlet desk) including papilla –verrucose (0), without appendage (1), with tuberculate and prickles (2), dome-shape papilla (3); NGRN (number of glochid row on nutlet edge) including lack of glochids (0), 1-row (1), 2-row.outer ones in a regular row (2); TD (tubercles on desk) including without spin (0), 2–3 spines (1), 4–5 spines (2), 5 spines (3); SE (surface emergence) including stellare-aculeate (0), veruucose-subverrucose (1), papilla- verrucose (2), Papilla (3), nonexpressiate (4); PS (prickles surface) including lack of prickles (0), verrucose (1), glossy (2)

To observe nutlets under SEM, they were mounted (two per each taxon and accession) onto standard aluminum stubs using double-sided adhesive tape and then photographed using a PHILIPS / FEI XL 20 Scanning Electron Microscope at 15 kV voltages. The measurements were based on 15–20 evaluations from each specimen.

The terminology used for describing the nine qualitative characters is in line with (Ma et al. 2010; Selvi et al. 2011; Yu et al. 2012; Hilger 2014). The very relevant traits were the shape of nutlet, the centerline of its shape, the base surface of prickles on the desk, lamella status, appendage on nutlet desk, the number of glochid row on nutlet edge, tubercles on a desk, prickles surface, and surface emergence (nutlet ultrasculpture) (Table 2).

Nutlet epidermal feature and its surface ornamentation (using SEM) are of diagnostic value in genera/species delimitation (Boyd 2002). The body of literature revealed their importance for delineating evolutionary pathways in the Boraginceae including Eritrichieae (Ovczinnikova 2007; Ovchinnikova 2008), Cynoglosseae and Eritrichieae (Hilger 2014), *Microula* (Yu et al. 2012), *Cynoglossum* L. (Akçin 2007), *Lithospermum* L. (Weigend et al. 2009), *Onosma* L. (Akcin 2008; Binzet and Akçin 2009), *Lappula* (Ma et al. 2010). Various nutlet traits were described and defined for these taxa. Reviewing the literature about our specimens paved the way for screening the final selected traits. For example, the traits related to the ornamentation of nutlet surface (states tuberculate and stellare-aculte of surface emergence) were defined according to (Ovchinnikova 2009) that studied the fruits of the Eritrichieae tribe. Moreover, the status of glochid and anchor was of great favor due to its significance concerning the Rochelieae tribe and particularly the *Lappula* genus. They were defined according to the study by (Ma et al. 2010) on nutlet dimorphism of the *Lappula* genus.

Finally, investigating the ontogeny and systematic importance of the fruits of Cynoglosseae and Eritrichieae (Hilger 2014) helped us achieving desirable characters and their status. These characters were scored as binary variables and numerical values, and then they were standardized and prepared for the following analysis.

The data were analyzed and examined by PCA, and WARD dendrogram using PAST software to study decimation of *L. microcarpa* and *L.barbata* species. Due to high morphological similarities between *L. microcarpa* and *L.barbata*, the Flora of Iran (Nasseh and Joharchi 2017) and *Flora Iranica* (Riedl 1996) were used for discerning them from each other.

### Phylogenetic analysis and tracing character evolution

The sequences for the internal transcribed spacer (ITS) region were obtained from GenBank (Table 3). Based on Weigend et al. (2009, 2013) and Gottschling et al. (2014), the nuclear ribosomal DNA internal transcribed spacer (ITS), as well as the chloroplast (cp) DNA regions tRNA-Leu (trnL) gene and trnL-trnF intergenic spacer, are appropriate for studying the phylogenetics (phylogeny) of the Boraginales at different taxonomic levels. Given the sequences deposited in the gene bank, we reconstructed the phylogenetic tree using ITS 1, 5.8S rRNA, ITS 2, of the nuclear region.

**Table 3:** List of taxa used in phylogenetic analysis with their GenBank accession numbers

Taxon	Tribe (based on Chacón et al. 2016)	GenBank accession number
*Heterocaryum macrocarpum*	Rochelieae	AB758300.1
*Heterocaryum szovitsianum*	Rochelieae	AB758298.1
*Heterocaryum subsessile*	Rochelieae	KU927721.1
Heterocaryum rigidum *	Rochelieae	AB758299.1
*Suchtelenia calycina* (1)	Rochelieae	LC194913.1
*Suchtelenia calycina* (2)	Rochelieae	LC194912.1
*Lappula balchaschensis*	Rochelieae	JX976776.1
*Lappula anocarpa*	Rochelieae	JX976775.1
*Lappula intermedia*	Rochelieae	JX976785.1
*Lappula patula*	Rochelieae	AB758305.1
*Lappula stricta*	Rochelieae	JX976798.1
*Lappula occidentalis*	Rochelieae	KU927723.1
*Lepechiniella wendelboi*	Rochelieae	AB758314.1
*Eritrichium rupestre*	Rochelieae	KU927644.1
*Eritrichium thymifolium*	Rochelieae	JX976807.1
*Eritrichium canum*	Rochelieae	KU927710.1
*Eritrichium splendens*	Rochelieae	JQ388501.1
*Eritrichium villosum*	Rochelieae	JQ388502.1
*Eritrichium pectinatociliatum*	Rochelieae	KU927713.1
*Eritrichium aretioides*	Rochelieae	KU927709.1
*Eritrichium nanum*	Rochelieae	JQ388499.1
*Hackelia micrantha*	Rochelieae	JQ388504.1
*Hackelia deflexa* (1)	Rochelieae	JX976808.1
*Hackelia deflexa* (2)	Rochelieae	KU927716.1
*Hackelia revoluta*	Rochelieae	KF849119.1
*Hackelia diffusa*	Rochelieae	JQ388503.1
*Rochelia macrocalyx*	Rochelieae	AB564700.1
*Rochelia bungei*	Rochelieae	AB564695.1
Rochelia disperma *	Rochelieae	LC410071.1
Lappula ceratophora *	Rochelieae	AB758301.1
Lappula semiglabra *	Rochelieae	AB758306.1
Lappula spinocarpos *	Rochelieae	AB758309.1
Lappula sinaica *	Rochelieae	LC410061.1
Lappula sessiliflora *	Rochelieae	LC410059.1
Lappula microcarpa *	Rochelieae	JX976788.1
Lappula barbata *	Rochelieae	LC410054.1
Asperugo procumbens *	Asperugeae	AB758290.1
Myosotis sylvatica *	Myosotideae	AB989064.1

* The species used for character evolution

To optimally show the intra-tribe relationship, the general topology of the phylogenetic tree was performed on the Phylogeny.fr platform (Fig. 2a) following bellow steps: (1) sequences were aligned via MUSCLE (v3.8.31) configured for the highest accuracy; (2) ambiguous regions were removed with Gblocks (v0.91b); (3) the phylogenetic tree was reconstructed using the maximum likelihood method implemented in the PhyML program (v3.1/3.0 aLRT) (4) the HKY85 substitution model was selected assuming an estimated proportion of invariant sites (of 0.435) and 4 gamma-distributed rate categories to account for rate heterogeneity across sites (the gamma shape parameter was estimated directly according to the data [gamma = 0.445]); (5) reliability for the internal branch was assessed by hiring aLRT test (SH-Like); and, (6) graphical representation and edition of the phylogenetic tree were performed using TreeDyn (v198.3) (Dereeper et al. 2008).

To study the character evolution (Fig. 2b), the sequences manually aligned via MUSCLE using MEGA software ver.7 (Kumar et al. 2016). Poorly aligned positions and divergent regions were eliminated by using Gblocks 0.91b, following the given options for less stringency (Castresana 2000). Phylogenetic analyses were performed using the combined 3-loci data set. The partitioned ML analysis was fulfilled using raxmlGUI 1.1 (Silvestro and Michalak 2012) under the GTR + G model with 1000 bootstrap replicates and with *Asperugo* (tribe Asprugeae) and *Myosotis (*tribe Myosotideae*)* chosen as out-groups. The evolutionary history of characters was traced over an ML tree in Mesquite 3.04 (Maddison and Maddison 2015). The ML approach was applied with the Markov k-state one-parameter (Mk1) model (Lewis 2001).

## Result

### General description of nutlet micromorphology

**Fig. 1:**
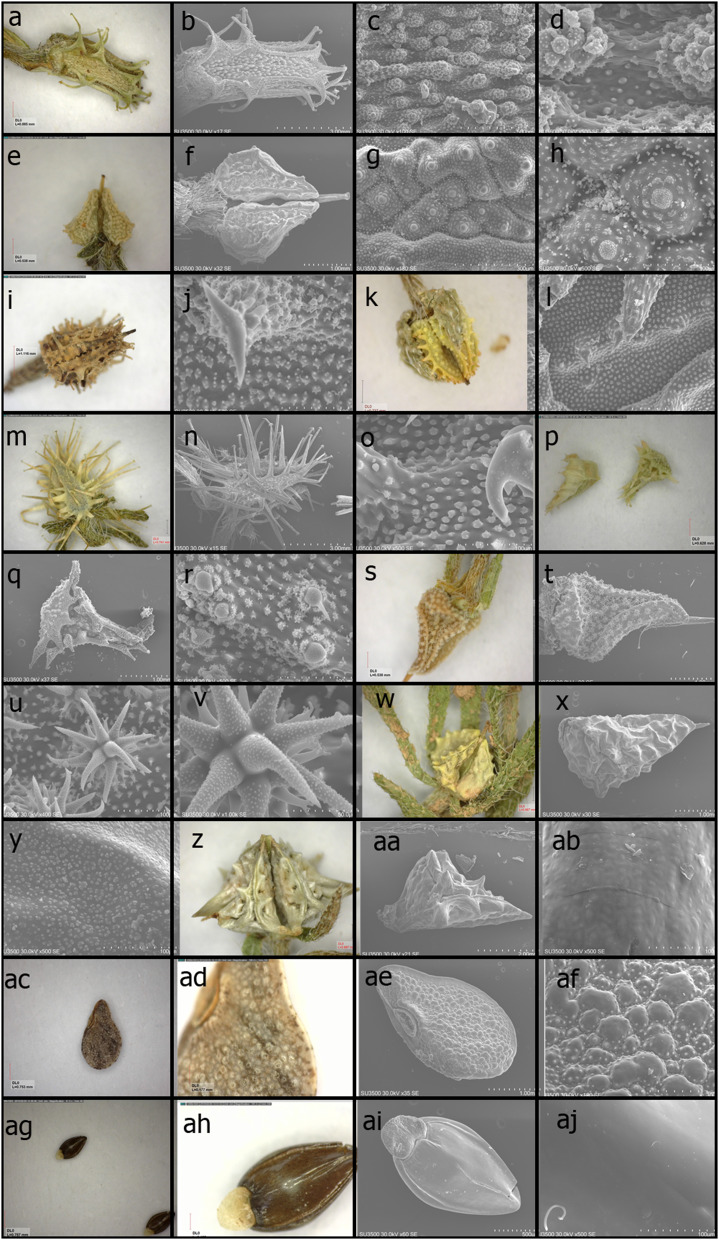
Six types of nutlet morphology in the studied species. **a**–**h = Type I; (a-d)** *Heterocaryum rigidum***; a**, **b:** An overview photograph of nutlet with stereomicroscope and SEM. **c**, **d:** The close-up views of the nutlet disk with “papilla verrucose with verrucae minutely muricate”. **(e–h)** *Pseudolappula siniaca*; **e**, **f:** An overview photograph of nutlet with stereomicroscope and SEM. **g**, **h:** The close-up views of the nutlet disk with “papilla with aggregate verrucose in the center”. **i-o Type II**. (**i**, **j)** *Lappula. barbata***; (k**, **l)** *Lappula. Microcarpa***;** (The more detail of these two species were described between different specimens in Fig. 3). *Lappula semiglabra* **m**, **n:** An overview photograph of nutlet with stereomicroscope and SEM. **o:** The close-up views of the nutlet disk. **p–v = Type III; (p–r)** *Rochelia sessiflora***; p**, **q:** An overview photograph of nutlet with stereomicroscope and SEM. **r:** The close-up views of the nutlet disk with prickles and verrucose on it. **(s-v)** *Rochelia disperma* **s**, **t:** An overview photograph of nutlet with stereomicroscope and SEM. **u**, **v:** The close-up views of stellare-aculeate emergencies in nutlet disk. **w-ac = Type IV; (w–y)** *Lappula spinocarpus***; w**, **x:** An overview photograph of nutlet with stereomicroscope and SEM. **y:** The close-up views of the nutlet disk with papilla with flowerlike verrucose on it. **(z–ab)** *Lappula ceratophora* **z**, **aa:** An overview photograph of nutlet with stereomicroscope and SEM. **ab:** The close-up views of the nutlet disk with papilla appear as a verrucose-like on it. **ac–af = Type V;** *Asperugo procumbense***; ac–ad:** the overview photograph of nutlet with stereomicroscope and SEM. **af:** The close-up views of the nutlet disk with papilla appear as dome-shape on it. **ag–aj = Type VI;** *Myosotis sylvatica***; ag–ai:** the overview photograph of nutlet with stereomicroscope and SEM. **aj:** The close-up views of the nutlet disk with a smooth surface

The nutlet's morphology and ultrastructure characteristics such as shape size, appendages, and surface sculpturing, varied among the studied taxa. The Rochelieae nutlets' shapes were different across samples; while some had ovoid shapes (ovoid, ovoid-triangular, and ovoid-rectangular), some other samples had pyramid shape (Fig. 1). In the out-groups, the shape of *Asperugo procumbense* was semicircular, and the *Myosotis sylvatica* was of ellipse shape. These two genera belong to Asperugeae and Myosotideae tribes, respectively.

Nine qualitative characters were selected for the morphological evaluation of nutlets. The results obtained from nutlet-ultrastructure investigations are described below and illustrated in Fig. 1. Generally, six different surface types were recognized among studied taxa based on nutlet ultrastructure characters:

#### Type I: *Heterocaryum* and *Pseudolappula* (Syn: *L. siniaca*)

There was no glochid or appendage on the nutlet disk, but a row of glochid (*Heterocaryum*) or glochid-like (*Pseudolappula*) on the nutlet edge. The glochids were distributed sparsely on the edges of the nutlets of *Pseudolappula*. The nutlet disk ornament of *Heterocaryum* was “papilla verrucose with verrucae minutely muricate” (called complex papilla), the nutlet disk ornament of *Pseudolappula* was “papilla with aggregate verrucose in the center”. It appeared that each of the microcapillaries found in *Psudolappula* were complex in the *Heterocaryum*, and each formed warts (verrucose), leading to a more complex and denser status.

#### Type II: ***Lappula*** (*L. barbata*, *L. microcarpa* and *L. semiglabra*).

Glochids in different sizes and rows can be seen at the nutlet edge and sometimes on the nutlet disk surface. Glochids had an anchor with 2–4 branches at the apex, and their surface was smooth. The ultrastructure of the nutlet emergencies was stellar-aculeate, and in some case the appendage, prickles, or tubercles could be seen (scattered or collected) on the surface of the nutlet disk and edge. The glochid stem was composed of fusiform cells, and there were tubercles with 2 to 5 mineralized spines on the stem. These tubercles were also present on the entire surface of the nutlet with a different distribution.

#### Type III: ***Rochelia*** (*R. disperma*, *R. sessiflora* = *L. sessiflora*).

The prickles were arranged in a stellate pattern and scattered throughout the surface of the nutlet. The surface of the prickles was not glossy and was of verrucose. The tubercles often had more than 2 spines, and the emergencies were stellar-aculeate (similar to type II). Nonetheless, the nutlet surface of *R. sessiflora* was similar to type II (presence of glochid on the nutlet edge). Moreover, the prickles and the accumulation of tubercles with more than 5 spines around each prickle shared more resemblance with type III.

#### Type IV: *L. ceratophora *and *L. spinocarpus*

There was not any glochid, tubercle, or prickles on the nutlet surface. The nutlet surface of *L. ceratophora* was not smooth, and the papilla appeared as verrucose-like. The ultrastructure of the nutlet in *L. spinocarpus* had a similar appearance to papilla with flowerlike verrucose. Also, the tubercles appeared as verrucose lacking any spines.

#### Type V: *Asperugo*

The nutlet surface lacked any glochid and prickles. Papilla appeared dome-shaped in different sizes, with verrucose at the base of it.

#### Type VI: *Myosotis*

The surface of the nutlet was smooth, and there was no ornamentation (nonexpressiate).

### Evolution of nutlet’s microstructural characters

**Fig. 2:**
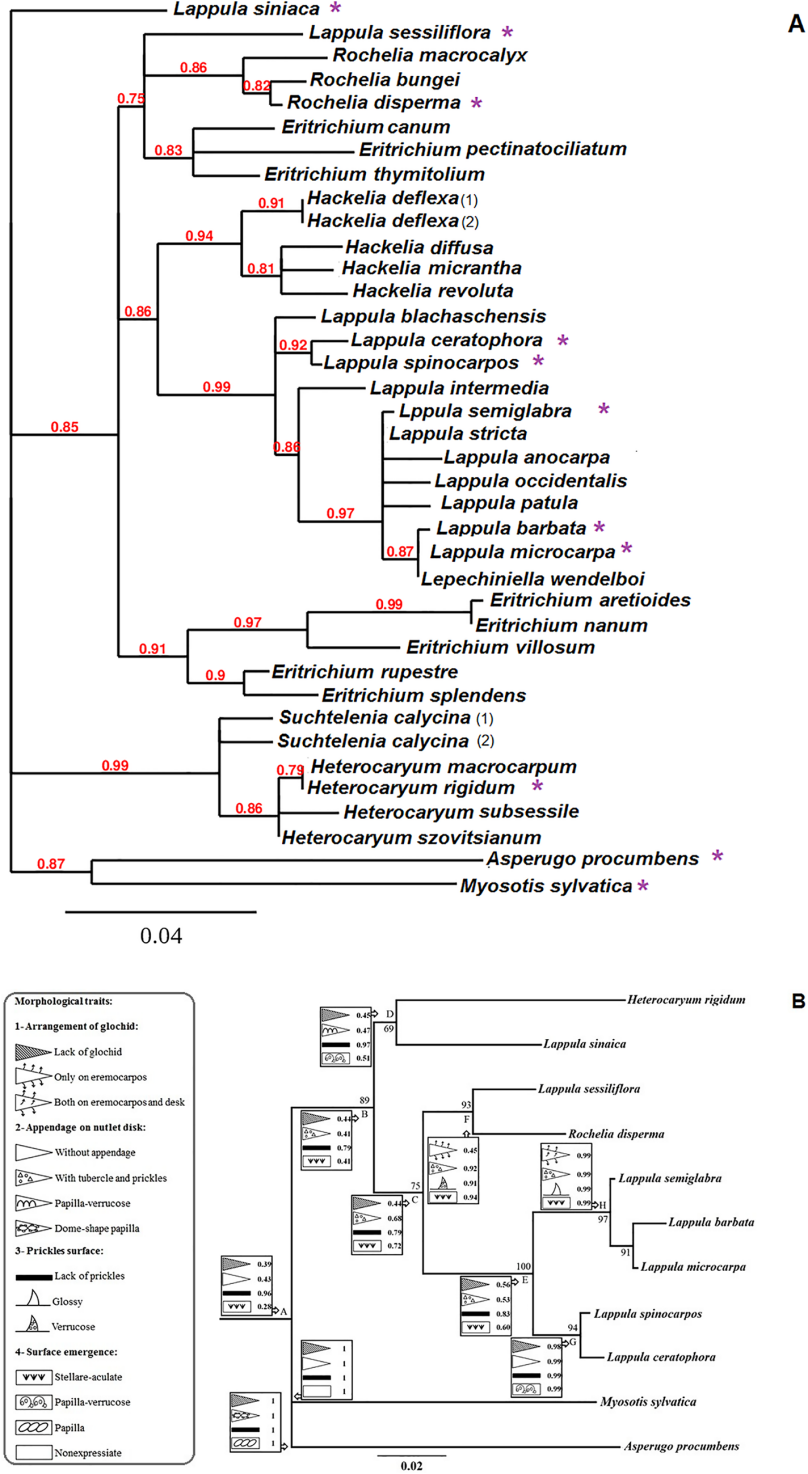
**A** Details of the ML tree of some species of Rochelieae showing the glancing view on phylogenetic relationships of different genera and species with focused on *Lappula* genus. The taxa selected for character evolution analysis were shown by an asteroid. **B** Results of nutlet character evolution are shown on the Maximum Likelihood tree of tribe Rochelieae (based on the internal transcribed spacer). Numbers on branches are Maximum Likelihood bootstrap support (only shown when ≥ 65)

We did not observe topological contradiction for the Rochelieae tribe in the analysis that were performed by constructing gene trees from the nr-DNA ITS, concatenated trnL-F–rpl32-trnL(UAG), and concatenated nr-DNA ITS–trnL-F–rpl32-trnL(UAG) data sets. This was consistent with what was reported by Khoshsokhan-Mozaffar et al. (2018). Therefore, for preventing sloppiness, we selected nr-DNA ITS because of better resolution. Details of the ML analyses based on the nr-DNA ITS data set confirmed the phylogenetic relationships of some species of Rochelieae and then formed a well-supported lineage encompassing two clades. This is totally in agreement with the topology published by Khoshsokhan-Mozaffar et al. (2018). One clade was composed of sub-tribe Heterocaryinae (*Heterocaryum*-*Suchtelenia*), with high support (BS = 0.99). The next clade contained most subtribe Eritrichiinae (BS = 0.85) comprising *Lappula*, *Eritrichium*, *Lepechiniella*, *Hackelia*, and *Rochelia*. Within this clade, *Lappula sinaica* formed the distinct branch and nested far away from other *Lappula* species. Moreover, *Lappula sessiliflora* was nested with a subclade of *Rochelia* species (Fig. 2A).

The resulting ancestral state reconstruction and the proportional likelihoods for character states are shown in Fig. 2B. Two out-group species *Asperugo* and *Myosotis* were unique with the bilaterally flattened and ellipse with a smooth surface, respectively. Tracing the evolution of nutlet micromorphology indicated that the glochids are not an ancestral character.

#### Arrangement of glochid character

The status of ancestral taxa (with or without glochids) was unclear, and the proportional likelihoods of all three characters were almost equal (node A). Transition to the glochids character occurred in the genus *Lappula* (*L. semiglabra*, *L. microcarpa*, and *L. barbata*) (node H).

#### The appendage on nutlet disk character

The status “without appendage” and “dump-shape papilla” in *Myosotis* and *Asperugo* (the proportional likelihood 1) differentiated these two tribes from each other and Rocheliea tribe. The tubercle and prickles on the disk were ancestral characters (the proportional likelihood 0.43). While the ancestor of these characters was unclear in node A, the status “tubercle and prickles on disk” was of more proportional likelihood in A and then C, D, F group. Transition to the “lack of appendage” status occurred in the *L. ceratophora*, *L. spinocarpus;* node G).

#### Prickles surface character

Tracing of character “prickles surface” showed the ancestral status of “lack of prickles” in node A that to be followed with less proportionality in nodes B, C, and D. Transition to the “glossy prickles” status occurred in the genus *Lappula* in node H. Moreover, the transition to the simple and complex “verrucose prickles” status was observed in genus *Rochelia* in node F.

#### Surface emergence

Tracing of surface emergence character was unclear in node A. Though, the proportional likelihoods of “stellar-aculeate” status had the highest node C ratio (0.72).

In node G, the transition to “verrucose-subverrucose” status (the proportional likelihoods 0.99) was stabilized as a synapomorphy.

Other traits were studied regarding evolutionary tracing that did not show clear evolutionary signals in the nodes, such as the shape of the nutlet, the lamella type, the shape of the nutlet, the lamella type, and the centerline of the nutlet disk.

### Close boundary of nutlet microstructures between *L. barbata* and *L. microcarpa*

**Fig. 3:**
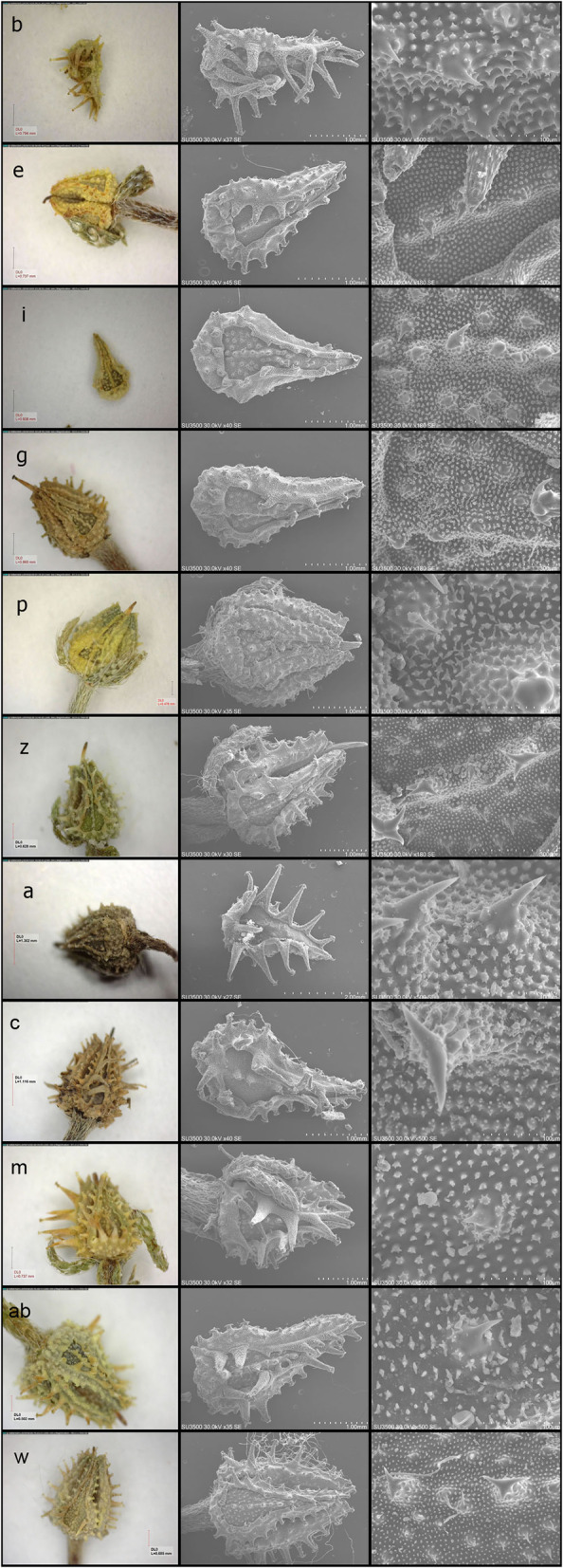
nutlet morphology in the different specimens of *Lappula microcarpa* and *lappula barbata*. The details of the studied specimens are in accordance with Table 1 (*L. microcarpa* codes: b, e, I, g, p, z, a*; L. barbata*: c, m, Ab, w)

**Fig. 4:**
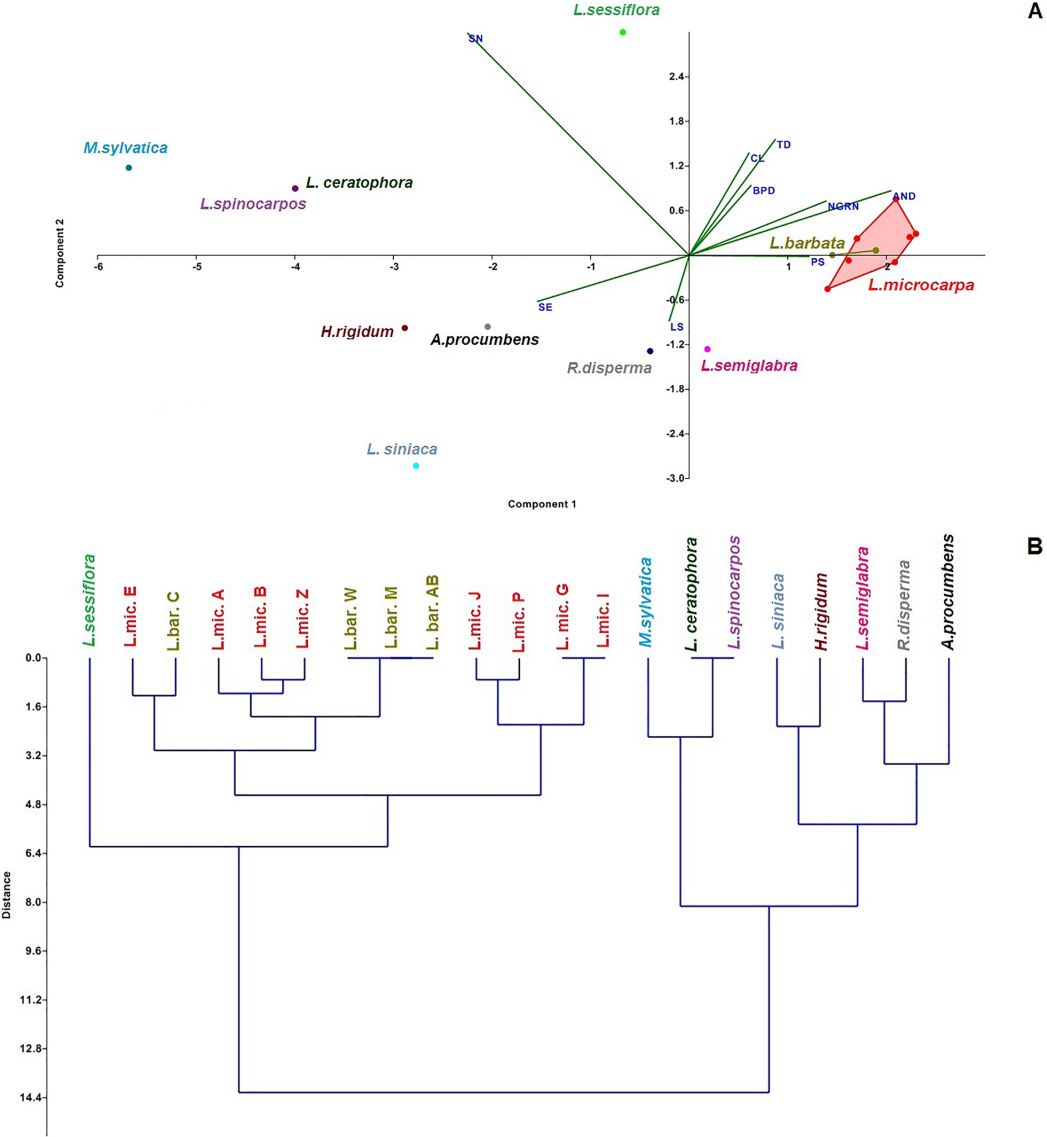
**A** PCA Biplot of nutlet characteristics of the studied taxa of Rochelieae tribe focuses on different specimens of *L. microcarpa* and *L. barbata*. **B** WARD dendrogram showing the relationship among different taxa focused on specimens belong to *L. microcarpa* and *L. barbata* based on nutlet characters. The codes are in accordance with Table 1. SN (shape of nutlet); CL (centerline of nutlet); BPD (base surface of prickles on the desk; LS (lamella status); AND (appendage on nutlet a desk); NGRN (number of glochid row on nutlet edge); TD (tubercles on a desk); SE (surface emergence); PS (prickles surface)

Both *L. microcarpa* and *L.barbata* had high micro-morphological similarities (Fig. 3). The very close relationship was studied by PCA analysis about other studied taxa in the tribe Rochelieae. In total, nine nutlet characteristics of the studied taxa were investigated (see nutlet SEM characters in Table 2). PCA analysis of nutlet characteristics revealed that the first two PCs comprised 75.83% of the total variability of the taxa. In the first PCA axis, characters such as the appendage on nutlet desk, shape of nutlet; surface emergence and prickles surface showed the highest correlation, while in the second PCA axis, other characters, such as seed shape of nutlet, tubercles on the desk and the centerline of nutlet showed the highest correlation. Therefore, these were the most varied micro-morphological characters of the taxa. PCA-biplot of micro-morphological characters (Fig. 4a) separated the taxa into distinct groups. All specimens of *L. barbata* and *L. microcarpa* were clustered together and close to *L. semiglabra* (Fig. 4a), while other taxa were grouped together. Moreover, *L. ceratophora* and *L. spinocarpos* overlapped each other.

Different clustering and ordination methods produced similar results; therefore, only the WARD tree of micro-morphological characters is presented here (Fig. 4b). The taxa were separated in the WARD tree of micro-morphological features, subsequently resulted in main branches. One included three genera arranged in two sub-groups: subgroup I, including all specimens, which belonged to *L.barbata* and *L.microcarpa*, while *L.semiglabra* diverged from others and placed in sub-group II. The rest of the taxa were clustered with each other in the same branch, similar to the results of the PCA plot (Fig. 4a). This result implies that the micro-morphological characters could not delimit two species *L. barbata* and *L.microcarpa*.

## Discussion

As in other Boraginaceae genera, the infrageneric taxonomic significance of nutlet characteristics in tribe Rochelieae were found to be evident through investigation employing stereomicroscope and SEM (Ovchinnikova 2009; Weigend et al. 2009; Selvi et al. 2011; Yu et al. 2012; Hilger 2014). In this study, we found that the appendages on the nutlet varied between different genera. Although the fruit type of Boraginaceae is relatively constant, the variation in nutlet ornamentation has quickly occurred in some tribes like Cynoglosseae sensu lato (including tribe Rocheliea) and Trichodesmeae (Cohen 2014).

It is not surprising that *Lappula*, one of the largest genera in Rochelieae, exhibits considerable diversity in nutlet characters. Recent molecular evidence suggested that *Lappula* is polyphyletic (Khoshsokhan-Mozaffar et al. 2018). In this regard, Khoshsokhan-Mozaffar et al. indicated that species of the *Lappula* genus are scattered across the Eritrichiinae clade and formed three distinct lineages. *Lappula sinaica*, as a new genus, was segregated from *Lappula* and established as genus *Pseudolappula* (Khoshsokhan-Mozaffar et al. 2018). Moreover, as shown in our study, a distinct generic delimitation based on nutlet characters alone could be detected for *Pseudolappula* (syn: *Lappula siniaca*). Nutlet micromorphology of *Pseudolappula* (syn: *L. siniaca*) provides valuable data in favor of separating it from *Lappula* genus; so that, the characters are “no glochid or appendage on nutlet disk” and the type of nutlet ornamentation including “papilla with aggregate verrucose in the center”. The features of disk ornamentation indicated a more close affinity of *Pseudolappula siniaca* to *Heterocaryum* than *Lappula* genus, especially in the evolution of microcapillaries. Current data are also in accordance with molecular phylogenies provided by (Khoshsokhan-Mozaffar et al. 2018) that transferred closely related species *L. sessiliflora* to *Rochelia* genus. This conclusion has previously been suggested by various studies (Khoush et al. 2010; Huang et al. 2013; Mozaffar et al. 2013; Rolfsmeier 2013; Weigend et al. 2013). Moreover, the flowers and nutlet features (two of them undeveloped) designated more affinity of the species to *Rochelia* genus than *Lappula* (Popov 1974). Our result stipulated the features like prickles and verrucose on the nutlet, and the accumulation of tubercles with more than five spines around each prickle showed more similarity to genus *Rochelia*.

The present study convincingly provided a clear distinction between two species *L. ceratophora* and *L. spinocarpus*, which belong to sect. *Sclerocaryum* (Riedl 1967; Ovchinnikova 2009), from other *Lappula* genus. The lack of glochid, tubercle, or prickles on the nutlet surface and specific types of nutlet ornamentation (papilla with verrucose-like or flowerlike-verrucose) characterized the clade that includes them regarding recent molecular phylogenetic analysis (Khoshsokhan-Mozaffar et al. 2018).

We identified four different types of nutlet surface ornamentation among taxa. According to our study and reported by others (Cohen 2014), the ancestral type is ambiguous for the family and Rochelieae tribe. Given the matrix of cpDNA-nr-DNA ITS and nutlet surface analysis, (Cohen 2014) indicated the smooth nutlets as ancestral for the clade that includes Boragineae and Lithospermeae tribes, and nutlets with glochids as a synapomorphy for Cynoglosseae sensu lato (including tribe Rocheliea). The results of the current study are in agreement with Cohen (2014) who demonstrated that glochid is not an ancestral trait but is a synapomorphy so that in node H, the transition trait occurred in the genus *Lappula*. Rocheliae is characterized by a transition from “without appendage” to “with tubercles and prickles” on the nutlet disk, and also by a shift from “lack of prickles” to “glossy prickles”. Also, a transition from the “nonexpressiate” status of surface emergence to “stellar-aculeate” status occurred in this tribe. Interestingly, in this tribe, smooth nutlets were a synapomorphy (Cohen 2014). Considering this, the transition to “lack of appendage” occurred in node G could indicate a synapomorphy of two species of sect. *Sclerocaryum* (*L. ceratophora* and *L. spinocarpus*).

In Boraginaceae, nutlets need to develop strategies to achieve dispersal ability. In previous studies, nutlets with glochids or wings have implied adaptive traits for additional dispersal types, such as epizoochory or anemochory (Ma et al. 2010; Selvi et al. 2011). The presence of glochid on the nutlet could explain the widespread geographic distribution of Cynoglosseae sensu lato (including Rochelieae) (Cohen 2014). According to (Weigend et al. 2016), two species of sect. *Sclerocaryum* applies “the whole-plant dispersal by wind or flash-floods” as a dispersal mechanism which could explicate the synapomorphy of “lack of appendage” observed in our results. Indeed, this mechanism caused the separation of nutlets from the mother plant to become unnecessary.

Morphologically, Rochelieae is characterized by divided calyx toward its base, hypocrateriform to infundibuliform corolla, pyramidal to subulate gynobase with 1–4 nutlets (Riedl 1967, Popov 1974, Chacón et al. 2016). However, *Lappula* species have blue to white funnel-form to campanulate corollas and form a scorpioid cymose inflorescence. In this genus, there are four homomorphic (sometimes heteromorphic) nutlets with one or more rows of marginal spines/glochidia (Riedl 1967, Popov 1974, Weigend et al. 2016). Morphometric results based on the nutlet morphology indicated four essential points, including the close affiliation of *L. sessiflora* to *Rochelia* than to the *Lappula* genus, the narrow barriers between *L.barbata* and *L.microcarpa*, and the apparent distinction of *L.siniaca* from other *Lappula* species. More affinity of *L.sessiflora* to *Rochelia* genus was proposed by Popov (1974), followed by different molecular phylogeny studies that finally ranked it as *Rochelia* species (Khoshsokhan-Mozaffar et al. 2018). Four zygomorphous and unequal nutlets were interpreted as diagnostic features of the *Heterocaryum*. These nutlets are small, flat, dorsiventrally compressed, with dentate wings (Khoshsokhan-Mozaffar et al. 2018). Although in the phylogenetic tree, the *Heterocaryum* genus did not indicate the close affinity to *Lappula siniaca*, morphometric results showed more similarity to *Heterocaryum* than other *Lappula* species. Based on morphometric results, *Lappula spinocarpos* and *L. ceratophora* (both of the sect. Sclerocaryum) were closely located to each other that showed more affinity to *Myosotis* and were apart from other *Lappula* species. These two species have nutlets with uniform, thick, and stonelike tubercles, with almost smooth surface. Although these two species have experienced different taxonomic treatment and have often been identified as distinct genus [*Sclerocaryopsis*; Sadat (1989)], recent morphological and molecular studies have identified them as members of the genus *Lappula* (Nasir 1989; Khatamsaz 2002; Chacón et al. 2016; Khoshsokhan-Mozaffar et al. 2018).

Nutlet micromorphology results did not show clear distinction among species *L.barbata* and L*. microcarpa*. The morphological complexities of these two species have already been addressed by different taxonomists (Popov 1953; Akhani 1998). A revision of the *Lappula* genus by (Nasseh and Joharchi 2017) suggested that the two species may be synonymous and further molecular studies need to conduct. Moreover, according to the molecular results (Khoshsokhan-Mozaffar et al. 2018) in the nr-DNA ITS tree of Rochelieae, the clade delimiting these two species was not well-supported. All of this evidence and our results convinced us to consider all specimens belong to *L.barbata* and *L.microcarpa* as “complex species” including *L.mocrocarpa*, *L.barbata, L.semiglabra* that could be comparable with *L.wendelboi*. Recently, *Lepechiniella wendelboi* Riedl was considered as a synonym of *Lappula wendelboi* (Riedl) Khoshsokhan & Kaz. Osaloo, based on molecular analysis (Khoshsokhan-Mozaffar et al. 2018). According to the literature, the three *Lepechiniella* species (*L. albiflora, L. persica* and *L. wendelboi*) were transformed to *Lappula* genus because of morphological and molecular similarities.

A supplementary molecular study and nutlet micro/macromorphology in different geographical regions is needed to synonym *L. barbata* and *L.microcarpa* or any taxonomical treatment.

The variety observed in the nutlet of *L.microcarpa* and *L.barbata* could be related to seed heteromorphism that formerly is known to occur in a few Boraginaceae genera, e.g. *Eritrichium, Lappula* (*L. duplicicarpa* and *L. semiglabra*), and *Heterocaryum* (Wang et al. 1989). In species with heteromorphic seeds, the variations in shape, color, size, or associated structures (wings or bracts) could be a strategy for flexibility in modes of dispersal, dormancy, and timing of germination. This could be helpful regarding the seedling establishment in arid regions (McEvoy 1984; Imbert 2002; Evans et al. 2007; Venable 2007; Sun et al. 2008; Wang et al. 2008). Zhao et al. (2008) reported that *Lappula duplicicarpa* and *L. semiglabra* have heteromorphism nutlets with long and short glochids. Given the close relativity of *L.semiglabra* to *L.barbata* and *L.microcarpa*, it is recommended that future studies focus further on the nutlet heteromorphism.

## Conclusions

In this study, the nutlet ultrastructure pattern of Rochelieae was systematically informative at the genus level, but not at the species level. The results showed that glochid is not an ancestral trait but is a synapomorphy and the transition to this trait occurred in the genus *Lappula*. Nutlet micromorphology results in this study provided no clear distinction between species *L.barbata* and L*. microcarpa*.

## Data Availability

The data used and analyzed for the current study can be obtained from the corresponding author.
